# Retrodisplacement of the Uterus*Published by request of Ellis County, Texas, Medical Society, July 14, 1908.

**Published:** 1908-08

**Authors:** Harry K. Leake

**Affiliations:** Dallas, Texas


### Original Articles.

For Texas Medical Journal.

### Retrodisplacement of the Uterus.*

BY HARRY K. LEAKE, A. M., M. D., DALLAS, TEXAS.

Recently I was asked by your president to read a paper before this society on retrodisplacement of the uterus. I shall attempt to comply with this request, notwithstanding that this subject has emerged so often from the crucible of critical discussion and analysis it would seem that there is little, if anything, left to be said. But, as happens with every other question in medicine and

*Published by request of Ellis County, Texas, Medical Society, July 14, 1908.

surgery, so here the perennial dissenter arises to confute the orthodox view and must be reckoned with in order that our therapeutics shall be justified by a careful revision of our practice.

In every text-book on gynecology uterine retrodisplacements are portrayed with the minutest detail and with numerous variations, but all based upon the well-known anatomy of the female pelvic organs, certain routine methods of treatment recommended for their correction.

Broadly stated, with respect to the treatment of uterine retrodisplacements, gynecologists might be divided into two classes. First, that which would correct every uterine retrodisplacement, whether discovered accidentally or otherwise with the intent of prophylaxis; and second, that which would treat the condition only when attended by complications. Concerning the first, it may be said that there is apparently sound argument which defends the view held, for it is a matter of common observation that the displaced uterus, although movable and without symptoms, may at any time become fixed by disease, or have its vascular and lymphatic circulation so deranged as to require a radical procedure for the cure of the patient, at a time, perhaps, when she is less able physically to undergo it. Upon these grounds, I think it was, that the late Dr. Henrotin, of Chicago, some years ago emphasized the prevalence of uterine retrodisplacement in women and advised treatment for the condition whenever discovered. Indeed, his advice carried with it the suggestion of routine examination of women to eliminate the presence of uterine retrodisplacement if for no other reason. Many such patients, single and married, live with the possibility of contracting gonorrhea or other infections of the pelvis which, by resulting adhesions, will convert the mobile uterus into a fixed organ with pelvic and constitutional distress. The same might be claimed for these infections when the uterus is in its so-called normal position, but the retrodisplaced organ, on account of the want of drainage and its disturbed vascular and lymphatic circulation, is more susceptible to the disease and less likely to recover when this has occurred. In fact, the soil for the implantation and growth of the infecting germs is prepared already for their reception and active development. Moreover, an operation now for the restoration of the uterus to its normal position, in addition to the removal of the diseased tubes and ovaries, usually means a firm fixation of the fundus to the abdominal wall, thus destroying its natural function of mobility which, although the organ will never be pregnant, might still conserve its remaining anatomy and physiology. Sterility, too, is a frequent accompaniment of the displaced organ which has been known to disappear on restoring it to its normal position. Women with retrodisplaced uteri for years without symptoms have become great sufferers from a sudden or gradual incarceration of the fundus between the tense lateral boundaries of Douglas' pouch, thus producing engorgement of the entire organ with pressure upon the rectum and the sacral plexus of nerves, causing pressure symptoms with dragging and weight in the pelvis, sacralgia and pains down the thighs with or without irritation of the bladder. If from other causes neurasthenia has preceded or follows the displacement, this is much intensified and made more difficult of cure. Such possibilities may affect the child-bearing woman, but assume less or no importance in the unmarried, where congenital retrodisplacement is sometimes found, but seldom, if ever, manifested by symptoms of any kind, that may never arise until the marriage state is contracted. Probably such cases have no place in the argument for prophylaxis in the treatment of uterine retrodisplacement; they had better be left alone, and the energetic efforts made by some to replace and hold in position these congenitally displaced and small uteri are ill-advised and often productive of the very worst consequences. In these subjects the retroversion of the uterus is often combined with retroflexion of the fundus, or the entire organ, normally anteflexed, occupies an extraordinary posterior position in the pelvic basin, the fundus not being felt above the pubis. Such positions and malformations are the result of an abnormal fusion of the Mullerian ducts in the process of the development of the uterus which in some degree may be arrested, but it is doubtful if symptoms can be traced directly to the condition, for such a woman may be considered normal and treatment to rectify them will usually be not only futile, but probably disastrous.

Excepting an attempt to replace a retroverted uterus to cure sterility, I desist from interference with the mobile retrodisplaced uterus which gives no subjective symptoms. In these circumstances "Sufficient unto the day is the evil thereof" is a reasonable and practical antithesis of "An ounce of prevention is worth a pound of cure." A careful history of a large number of cases will discredit the professional Don Quixote with his unnecessary and perhaps hurtful zeal in subjecting these patients to local and surgical measures that engraft foul vaginal discharges, erosions and ulcerations, abeyance of the marital relation, impairment or complete obliteration of uterine mobility so necessary to the normal function of the organ, upon an inoffensive, if somewhat abnormal position of the uterus. In refutation of an opposite view expressed by Dr. Hancock, of Norfolk, Va., who apparently argues for the prophylaxis side of the matter, Dr. Byron Robinson, of Chicago, timely discusses the subject. If the statements of these disputants be taken as a fair index of the whole controversy regarding the pathology and treatment of retrodisplacement, I see no logical reason for such contention which in a final analysis turns upon the presence or absence of complications attending the displacement. These, consequently, seem to be the crux of the matter in dispute. What are complications? And assuredly these can not exist without giving rise to symptoms. It is common knowledge that many retrodisplaced uteri are revealed only by accident; when symptoms are evident, complications may be assumed and will be demonstrated. The uterus weighted backward by a fibroid or buffetted between an extraneous tumor and the sacrum causes symptomatic pressure that is felt chiefly in the sacral nerves and their irradiations. Likewise, the uterus is drawn backward and fixed in the sacral hollow by adhesions, the end products of pelvic inflammation of various origin. The symptoms of the condition lie principally in the tumor pressure or adhesions. These are the *corpus delicti* which, on being removed, the uterus is released with an improved mobility and circulation. Such complications are admittedly indications for their removal jointly with measures for restoring the displaced organ to its normal position and, if possible, to do this without abolishing its mobility. Dr. Robinson rightly lays stress upon the latter as the normal *sine qua non* of uterine structural health and function. But even with mobility a retrodisplaced uterus may cause symptoms that will prove a source of pain or invalidism. In certain cases the venous and lymphatic circulation may be obstructed, causing retroactive engorgement of the uterus with derangement of its normal metabolism. It is true that in the normal condition the latter seldom is stable, owing to the demand of the several functions of the uterus, but this instability is delicately provided for by the free vascularity of the organ, the veins being so disposed as not only to retain an abundant quantity of blood in the walls of the uterus according to its various needs,—such as pregnancy and menstruation,—but to allow different positions of the uterus without obstructing its circulation. However, a congenital defect may co-exist and particularly in major degrees of displacement the veins in the broad ligament may become stretched sufficiently gradually to impede the flow of blood through them to such a degree as to entail engorgement of the uterine tissues with symptoms that demand treatment for their relief. This stretching is initiated and aggravated by the persistent backward position of the uterus restricted within a limited arc of mobility; practically, it is a fixed organ, "pinned down behind the intestines," making pressure on the sacral plexus of nerves, with dragging and weight sensations in the pelvis through congestion of the entire uterine body. Hence, this vascular derangement is a complication deserving relief notwithstanding the uterus is not fixed immovably in retrodisplacement—its position is abnormal with symptoms in contradistinction to a large class of patients where retrodisplacement is present without symptoms as in the cases heretofore mentioned.

It has been claimed that on account of its wide range of movement the uterus has no definite—consequently normal—position in the pelvis, otherwise it would be a fixed organ. This statement is a juggling with terms. The fundus periodically is at rest behind the pubis, from which it revolves backward, but the opposing resiliency of the broad ligaments and the upward drag of the utero-sacral ligaments on the cervix tend to keep the longitudinal axis of the organ at nearly a right angle with the vagina—a provision of nature against prolapse. Therefore, its position is definite, not only as respects its temporary resting place behind the pubis, but its intrinsic tendency to resist mobility in a backward direction. Were the organ fixed at any point along its semi-circular, eccentric arc of mobility, particularly to the abdominal wall, interfering possibly with its symmetrical expansion in pregnancy, this impairment of function might affect it seriously as a child-bearing organ; but fixation followed over the anterior ninety degrees of its arc of revolution—although in contact with the abdominal wall—is attended by less evil consequences than when this occurs over the remaining posterior ninety degrees of its arc of normal mobility. As already hinted, the circulation of the uterus is adapted normally for a forward position of the organ with its temporary variations, but fixation does not obstruct it as when this occurs with the organ in a retrodisplacement. Therefore, a fixation forward is a choice or evils, operating with less detriment than the morbid fixation in retrodisplacement. Although somewhat lifted out of its natural habitat in the pelvis and its mobility destroyed, both logic and experience in many cases of retrodisplacement decide in favor of ventral fixation, sometimes even in the child-bearing woman.

Suspension of the organ by the various methods recommended can not be employed always successfully. Large, heavy and subinvoluted uteri will not remain in suspension in a great number of patients, while after the menopause, if replacement be indicated, fixation is less to be avoided. These chronically enlarged uteri usually are in a degenerate condition with a sluggish circulation aggravated by their retroposed position. This finally leads to painful engorgement, chiefly of the posterior wall, endometritis with or without adenomatous growths of the endometrium, menorrhagia, and predisposition to abortion; to permanently cure all of which a forward position of the uterus must be maintained and this is seldom to be accomplished by any procedure short of the ventral fixation. Of course, this dictum applies only to those cases wherein the enlarged organ can not be reduced in size and weight by depletion, curettage and tamponage preliminary to its forward reposition by surgical means. Lessened size and weight may demand happily the operation of election—some form of reliable suspension which now resumes its pre-eminence in the treatment of retrodisplacement. Likewise, after such preliminary treatment, if indicated, the position of the uterus with a limited mobility may be readjusted by a selected and skillfully applied pessary, but with slight promise of stable results after its discontinuance. Munde, who made a close study of these results, could furnish about 2 per cent of permanent cures—a statement that accords with my own experience. Some English gynecologists, I think, unwarrantably place the figures as high as 30 per cent.

Theoretically, at least, a failure with aggravation of the retrodisplacement is to be expected from the pressure and extension of the utero-sacral ligaments exerted by the upper bar of the pessary—the mechanical action upon which depends the apparent good result of the instrument. Moreover, the pessary is applicable in strictly retroversion cases. Retro-versio-flexed uteri, as a rule, are not amenable to this treatment, often being made worse through increase of the angle of flexion, attended by further lowering of the fundus. After labor, however, should there be a tendency to retrodisplacement—which happens frequently—pessaries are required urgently, and if employed early will be followed by gratifying results. Good involution is favored by the action of a suitable pessary, following which the instrument may be removed. Large rubber rings are best suited for the purpose, to be replaced later by a properly fitted hard-rubber instrument.

Ventral fixation, as performed ordinarily, is unsatisfactory. Complications, if present, having been removed, the uterus is drawn upwards to the small abdominal incision. Through-and-through sutures of silk-worm gut, including a safe hold of uterine tissue above and below the junction of the tubes with the uterus, are passed and tied. The remaining portion of the incision is closed in the usual way. The subsequent union is peritoneal, and can not be depended on for permanent fixation, the uterus under some strain breaking away, or the adhesions being absorbed or unduly extended finally, there is recurrence of the retrodisplacement. Herman, taking his cue from the operation of colotomy, where peritoneum is joined to muscular structure, inverts the peritoneum on both sides of the uterus, which is drawn well into the abdominal incision and closely applied to the borders of the exposed recti muscles. The sutures are then adjusted in the conventional manner just mentioned. Strong and durable adhesions may be expected, and in the many cases where I have employed this method of Herman, its superiority has been demonstrated.

In suspending the uterus the round ligaments are employed almost universally. These ligaments have no function either as supports to the uterus or as agents maintaining the organ in a forward position; they are vestigial remains of variable size and strength; but by shortening they may be utilized to keep the uterus in anteversion. The Alexander-Adams operation seeks to accomplish this by retrenching and anchoring their thinned-out extremities near their attachments at the external inguinal rings. Objections to this method are, the occasional difficulty in finding the ligaments; their attenuated ends, which will not endure strain or weight; the possibility of suppuration within the inguinal canals following traumatism and suturing, and that may enter the abdominal cavity causing a serious, if not fatal, peritonitis. More than this, the operation is available only where there is positive evidence of absence of intra-abdominal complications—a differentiation frequently unsupported by the results. The method of Goldspohn, which attempts to deal with complications through the surgically enlarged inguinal canals and rings, need be mentioned only to be condemned. To avoid every possible element of failure, it seems wise to do the operation through a small median incision, which may be extended to meet all requirements.

The mechanism of shortening the round ligaments varies with the conception of many operators who extol their own procedures; to describe all of them appears superfluous. A loop of each ligament, sufficiently long to lessen its curve, and thus approximate the uterus to the pubis, is made permanent by sewing its borders together with catgut or fine silk. These loops may be laid and sewed upon the remaining portions of the ligaments or upon the anterior face of the uterus; or being carried through a perforation in the broad ligament are joined by a suture to the posterior wall of the uterus for which a kind of sling is formed. The loops simply may be sewed to the abdominal wall a short distance outside the abdominal incision. With the exception of the last method which fixes the loops upon some exterior structure, and so has the advantage of suspending the uterus by the stronger ends of the round ligaments, there is probability that the sustained weight will extend gradually the smaller ends and finally allow intestinal coils to lie upon the anterior uterine wall. This additional weight, *plus* the effect of intra-abdominal pressure, in coughing, straining and other physical efforts, proves a *coup-de-grace* of the multiplied forces which re-establish the retrodisplacement as a frequent sequel of this plan of uterine suspension. Some operators have exsected deliberately portions of the ligaments and fixed the distal ends by suture upon the sides of the uterus, or by means of a perforation of the broad ligaments grafted the cut ends upon its anterior wall. The method is objectionable in that the weaker ends of the ligaments are used for the suspension which may fail also by reason of the insecurity of the artificial junction of the ligament with the uterus. Recently a form of suspension has been attempted by elaborate quilting of the broad ligaments on their anterior aspects.

Immediately following the announcement by Gilliam of his method of shortening the round ligaments by drawing loops through perforations in the recti muscles about an inch away from the abdominal incision and securely fastening them on the surface of the aponeurosis, I began to do this operation and have employed it with the greatest satisfaction in all suitable cases. Its advantages are obvious. The stronger uterine ends of the ligaments, forming one limb of each loop which takes up the slack, are utilized no less than the weaker ends to swing the uterus from a fixed point in the abdominal wall and yet allow sufficient mobility of the organ in the performance of its several functions. The claim that intestine might fall into the vacant space between the fundus and the abdominal wall and become imprisoned has no justification in my experience or in that of others, but such possibility may be anticipated by obliterating this space with a few fine sutures uniting the peritoneum to the fundus when closing the abdominal incision. The union by these sutures will offer little, if any, opposition to the future movements of the uterus. Besides affording some support to the latter until the ligament loops have become united firmly with the separated fibres of the recti muscles and aponeurosis to which the fixation has been made.

We are mindful of the fact that in all suspension operations by means of the round ligaments the important function of the utero-sacral ligaments and their relaxed condition in retroversion are somewhat, if not entirely, ignored. Nevertheless, it appears that if the fundus is kept forward for an indefinite period as effected by the suspension operation, this relaxation disappears and the ligaments regain their normal tone. However, to expedite or make certain the latter, they have been shortened by the vaginal route, or as advocated by Stoner and others, intra-abdominally. The combined operation of shortening both the round and utero-sacral ligaments within the abdomen has been carried out and this operation may prove to be the *ultima thule* of the future. In my judgment, all vaginal operations for shortening ligaments or fixing the fundus may be neglected safely.

Strictly speaking, there is no medical treatment for the pathology of clinical retrodisplacement. The evil consequences *per se* of the condition can be remedied only by surgical means. But it should be remembered that pelvic disease, with which retrodisplacement must be classified, is bound up often with constitutional derangement that discovers its own original cause. Pelvic disease of an aggravated and persistent type may originate and intensify a nerve depression that may impair seriously the well-being, or even threaten the life of the patient. But to refer every perturbation of the nervous system to a reflex from some minor pelvic ailment or abnormality, such as a latent retrodisplacement, is an error both in philosophical experience and therapeutic results. It may be shown justly that the most serious types of neurasthenia are not associated always as cause and effect with any form of retrodisplacement. The latter, if causing hemorrhage, endometritis, dysmenorrhea, constipation and bladder irritation will still further lower the nerve tone that has been depressed primarily by other causes, and, if possible, should be corrected. But the final cure of the patient awaits general treatment nicely adapted to remove other factors that involve a problem made confusing and intricate by the existence of a retrodisplacement. Therefore, the cure of the displacement is a part only, if an important part, in securing the ultimate welfare of the patient, but to focus the mind upon this feature of the case alone is a pathological obscession, escaped by the discriminating physician. At the last meeting of the

German Medical Congress, Lenharz, speaking on the relation of pelvic to general conditions, emphasized these facts, which, I submit, so far as retrodisplacement is concerned, have not received their real import since the views of Graily Hewitt were disproved, notwithstanding the protest of Mathews Duncan and his illustrious pupil, Ernest Herman, the eminent English clinical gynecologist. On the other hand, to operate every woman having a retrodisplacement without special or general symptoms, must be considered an exploit of professional graft for revenue or reputation or both that appeals more to the heedlessness of the physician than to his right reason and conscience.

